# Security Enhancement Using Cache Based Reauthentication in WiMAX Based E-Learning System

**DOI:** 10.1155/2015/878327

**Published:** 2015-08-17

**Authors:** Chithra Rajagopal, Kalaavathi Bhuvaneshwaran

**Affiliations:** ^1^Department of Information Technology, K. S. Rangasamy College of Technology, Thiruchengode, Namakkal, Tamil Nadu 637 215, India; ^2^Department of Computer Science and Engineering, KSR Institute for Engineering and Technology, Thiruchengode, Namakkal, Tamil Nadu 637 215, India

## Abstract

WiMAX networks are the most suitable for E-Learning through their Broadcast and Multicast Services at rural areas. Authentication of users is carried out by AAA server in WiMAX. In E-Learning systems the users must be forced to perform reauthentication to overcome the session hijacking problem. The reauthentication of users introduces frequent delay in the data access which is crucial in delaying sensitive applications such as E-Learning. In order to perform fast reauthentication caching mechanism known as Key Caching Based Authentication scheme is introduced in this paper. Even though the cache mechanism requires extra storage to keep the user credentials, this type of mechanism reduces the 50% of the delay occurring during reauthentication.

## 1. Introduction

WiMAX networks provide broadband wireless access over a distance of 50 KM with fixed subscriber station and over a radius of 5 KM to 15 KM with mobile station [1, 2]. Multicast and Broadcast Services of WiMAX make it suitable for E-Learning applications.

In the WiMAX based E-Learning system, the users can use the system anywhere and anytime. The E-Learning users are connected with the Base Station. The Access Service Network which comprises a group of base stations is interconnected and controlled by Access Service Network Gateway. The functions of the Access Service Network (ASN) Gateway include caching of E-Learning user profiles [2] and routing of data to the selected Connectivity Service Network (CSN). The ASN gateway also enables the E-Learning users to connect with the E-Learning server through the Connectivity Service Network. The Connectivity Service Network provides Internet connectivity to manage the sessions of E-Learning users through multicast group management functionality. Authentication, Authorization, and Accounting Server in Connectivity Service Network of WiMAX is responsible for authenticating the E-Learning users.

The open source E-Learning system such as MOODLE [3] is susceptible to session hijacking problem. E-Learning system discussed in [3] is also susceptible to man in the middle attack. The Personalized E-Learning system using SOA [4] does not address the security of the web services. E-Learning service using multiple biometric mechanisms [5, 6] addresses the initial authentication of users and does not focus on the security issues during the session management. The user name and password based profile questions are addressed in [7] to improve the authentication in online examination. However, the system is susceptible to security issues such as session hijacking. Intrusive and low-resources intensive approach [8] based on student verification to detect presence of the student does not address the need to perform verification during the intermediate session. The authentication protocol for E-Learning in [9] forces the user to perform full authentication process at every time of reauthentication.

In order to overcome the security problems in E-Learning systems the users are forced to perform reauthentication. When the users are forced to perform reauthentication process, the user credentials are verified and authenticated for a short period of time so the integrity of the application is preserved. The frequent reauthentication process introduces delay in accessing the application. An authentication protocol which performs reauthentication process with minimum delay is needed.

In this paper, cache based authentication protocol is proposed to support the authentication process with reduced delay. The user credentials during the initial authentication are cached at the Access Service Network Gateway to support faster reauthentication.

This paper consists of the following sections, Section 2 contains the proposed system architecture and Section 3 provides information about the system performance. In Section 4 conclusion is discussed.

## 2. Proposed System Architecture

**Figure 1:**
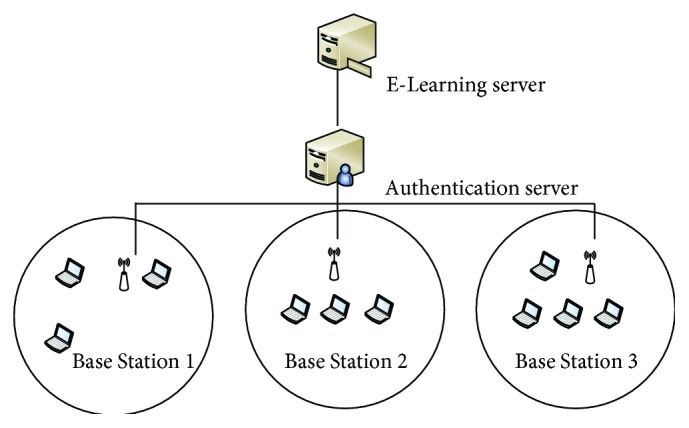
Proposed WiMAX based E-Learning system.

In the proposed WiMAX based E-Learning system, the Connectivity Service Network of WiMAX provides the Internet connectivity to connect with the E-Learning server. The users of the E-Learning system are connected with the Base Station using subscriber station.  Figure 1 represents the proposed architecture of E-Learning system using WiMAX.

Every user must be authenticated before accessing the WiMAX network. The Authentication Authorization Accounting (AAA) Server is responsible for authenticating the user. Initially the E-Learning users forward the username, password, nature of the service, duration of the needed service, and subscriber station MAC address as the initial attributes for authentication. The AAA server of WiMAX verifies these attributes and authenticates the E-Learning users by providing the Authentication Key (AuK), Session Key (SK), and Session Key Lifetime (SK_life_) using Session Based Authentication Protocol. These attributes are cached at the ASN Gateway controlling the appropriate Base Station.

To improve the security in WiMAX based E-Learning system, the users of E-Learning systems are forced to perform reauthentication after a predetermined period of time. The reauthentication process requires frequent message transfer and repeated authentication process between the same user and the authentication server. To support fast reauthentication, the information such as mobile station MAC address, Base Station MAC address, Authentication Key, Session Key, Lifetime of the Session Key, and the unique identifier SKID is cached at the Access Service Network Gateway during the initial authentication. In the reauthentication process the user sends reassociation requests with its Session Key, SKID along with the timestamp of authentication to the Base Station. The Base Station forwards the request to the ASN gateway. The ASN gateway generates the Session Key Identifier (SKID_new_) using the user information cached at its location and verifies the generated Session Key Identifier (SKID_new_) with the SKID in the reassociation request. During the calculation of Session Identifier the timestamp of authentication is included as one of the attributes.

When the session key identifiers are identical, the user is allowed to communicate with the ASN gateway by sending reassociation request. The ASN gateway verifies the type of the user along with the nature of request. If the request is from the existing user, new Session Key is generated and communicated to the user using Session Key update message. This key update procedure requires only four-way handshake message. Simultaneously the timestamp representing the time of reauthentication is updated in the cache.

The Session Key Identifier (SKID) for each E-Learning user can be calculated by applying the Secured Hash Algorithm (H) with the attributes represented in the following equation:

(1) $$\begin{array}{c} \text{SKID}=H\left[\;\text{SK}\left|\;\text{MAC}\_\text{BS}\;\right|\text{MAC}\_\text{SS}\left|\;U\_\text{ID}\;\right|TS\;\right]. \end{array}$$

In (1), SK is Session Key of the user, MAC_BS is the MAC address of the Base Station, MAC_SS is the MAC address of the subscriber station used by the E-Learning user, U_ID is the identity of user, and Ts is the timestamp at which the user is authenticated.

The usage of timestamp provides the additional identity of the user. A malicious user requesting the reassociation service does not possess the time at which it is authenticated, so the system is protected from the man in the middle attack. The reassociation request is initially handled by the ASN gateway of WiMAX network, so the unnecessary reauthentication requests from the users are discarded at the ASN gateway, protecting the E-Learning system from Denial of Service Attack.

The proposed authentication protocol performs fast reauthentication process with minimum number of message transfers between the E-Learning user and the authentication server using four-way handshake messages.

**Figure 2:**
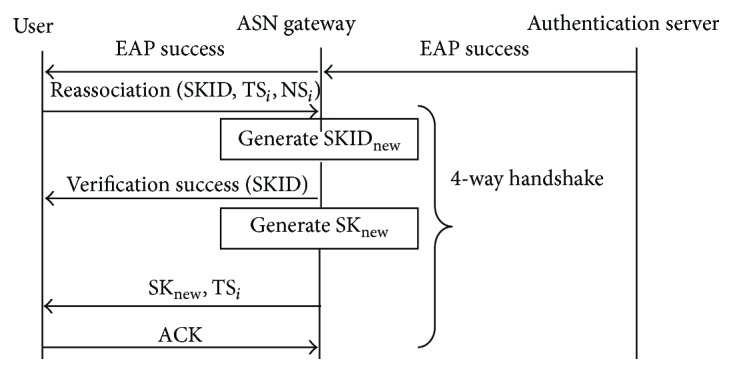
Handshake messages in reauthentication process.

The four-way handshake message is used to update new Session Key to the E-Learning user during reauthentication process.  Figure 2 represents the message exchanges during reauthentication process with the four-way handshake.

During reauthentication the ASN gateway is responsible for verifying the user and after identifying legitimate existing user, new Session Key is updated to the user along with the timestamp representing the time at which the reauthentication is performed. Thus, the delay during the reauthentication process is reduced. The repeated reauthentication process protects the system from session hijacking problems where another user impersonates and utilises the service. The proposed Session Based Cache Scheme enabling authentication is presented in the following section.

### 2.1. Session Based Key Caching Scheme

In this section, proposed caching mechanism at ASN gateway for rapid reauthentication of E-Learning users is discussed. This type of caching technique can be used with fixed E-Learning users connected with the Base Station. In this scheme the user information exchanged during the initial authentication is cached at the ASN gateway followed by forwarding the request to the authentication server. During the reauthentication process, the user verification is done at the ASN gateway and it is used only for updating the Session Key. This caching scheme reduces the number of redundant message transfers between the E-Learning user and the authentication server at the time of reauthentication.

The attributes used in the algorithm are as follows:

 
*C*_*i*_: context information about user identity,
 SKSA_*i*_: security information of the E-Learning user_*i*_ such as session identity and Authentication Key,
 SID: identity of the session,
 Session_life_: duration of the session,
 Count: number of times at which the reauthentication is performed
 ASNG_ci_: cache available at the Access Service Network Gateway_ci_,
 AS: Authentication, Authorization, and Accounting Server,
 AT: authentication table, representing the authentication table available at the server with each entry representing the user information,
 TS_*i*_: timestamp, representing the time at which the user_i_ is reauthenticated,
 Session_timeout_: expired session due to maximum number of reauthentication processes.

The functions used in the algorithm are as follows.

*Cache_Notify (C*_*i*_ *, SKSA*_*i*_ *, SID, Session*_*life*_, TS_*i*_). At the time of initial authentication this message is used by authentication server to maintain the cache maintenance at ASN gateway. It enables caching of information such as user identity, security association information, session information, and time at which the client is authenticated.

*Insert_Cache (C*_*i*_ *, SID, Session*_*lifetime*_ *, ASNG*_*i*_, TS_*i*_). This message is issued after the successful reauthentication, to update the authentication time and count attributes at the cache and also in the authentication table.

*Cache_Update (ASNG*_*i*_ *, C*_*i*_ *, SID, Session*_*life*_). This message is issued when the cache is full. The session entry with least duration of session is replaced with the new entry. This message is cascaded with the AT_Update message to update the corresponding entry in the authentication table to avoid the future reference to the same cache entry.

*Delete_Session_Entry (C*_*i*_ *, SID, Session*_*timeout*_). This message is periodically issued to improve the cache hit ratio, by deleting the expired session in the cache.

*AT_Update (C*_*i*_ *, SID, Session*_*timeout*_). This message is used to update the user information in the authentication table maintained at the authentication server. After the receipt of the message the server performs the initial authentication even when the already authenticated user sends the reauthentication requests.

The proposed cache based authentication algorithm is represented in Algorithm 1.

In this algorithm at initial network entry time, E-Learning user identity is verified and acknowledged with authentication and Session Key, Traffic Encryption key using AAA server. So the new user can use the services of WiMAX network for E-Learning. When the same user requests reauthentication the ASG gateway verifies the user identity and forwards the updated Session Key and timestamp TS_*i*_ to the user.

### 2.2. Least Session Based Cache Replacement Algorithm

In the Least Session Based Replacement Algorithm (see Algorithm 2), the entry in the cache which has least time of expiry in the session duration is evicted from the cache and replaced with the new user entry. The session replacement is done using the Cache_Update message generated by the corresponding ASN gateway. For maintaining the consistency between the server and the cache, the messages discussed in the previous section are used.

E-Learning user credentials are to be replaced efficiently so that the cache hit during reauthentication can be improved. When the cache hit ratio increases, the latency in reauthentication is reduced. In the Least Session Based Cache Replacement Algorithm the cache items with maximum number of access trails for reauthentication process are replaced with new user credentials. The users of the session with least lifetime may not request reauthentication. The cache entries with minimum active lifetime are also selected for eviction using the Session_life_, counting attributes in the proposed cache replacement algorithm.

## 3. Performance Evaluation

The proposed system is simulated with the NS-3 simulator. The simulation is performed with different traffic loads. The request for authentication can be from different categories of the E-Learning users such as for online learning, e-seminar. The performance of the system is evaluated based on latency in authentication, latency with various cache sizes. The latency in authentication is evaluated by simulating the system with three hundred E-Learning users with varying numbers of base stations and also with a cache size of 200 GB. The system with different cache sizes is also simulated with three hundred E-Learning users.

The experiment is conducted with the minimum duration of three hours per session consisting of six modules. The maximum duration of each module is designed with thirty-minute duration [10]. So the allowable limit for reauthentication is calculated using the following mathematical model.

Let *δ* be the probability that a user requests reauthentication before the session expires.

Let *T*_*n*_ be the time at which the user request for re-authentication.

Let *T* be the duration of the session.

Let *T*_*p*_ be the session cache period.

Using the residual life theorem [11], the *T*_*n*_ has the exponential distribution with fixed duration of the session *T* with the duration as 0 ≤ *T*_*n*_ ≤ *T*; then *δ* is represented as

(2) δPTn≤Tp=∫Tp=0T1T∗∫Tn=0Tpμe−μTndTndTp=∫Tp=0T1T1−e−μTpdTp=1TT+e−μT−1µ.

For the duration of three hours the probability that a user can be successfully reauthenticated using the cache is represented with Table 1.

Table 1 represents that the six reauthentication processes of E-Learning consisting of three-hour session can be handled effectively. So in the proposed algorithm the number of times at which the reauthentication can be handled (count) is taken as six.

### 3.1. Authentication Latency

**Figure 3:**
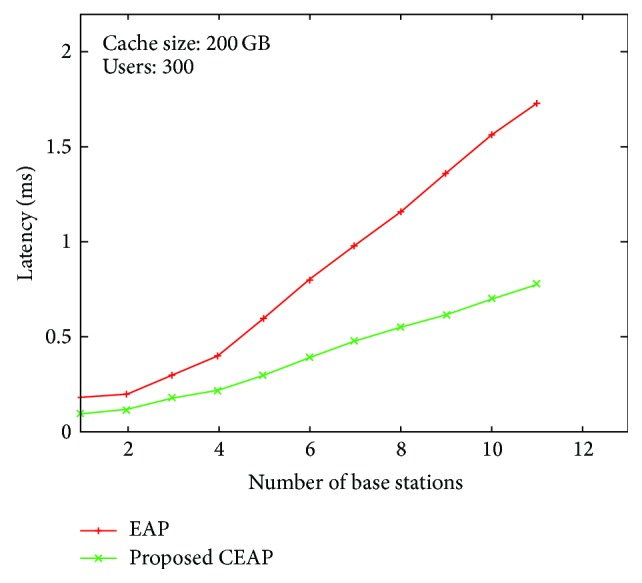
Authentication latency.

EAP based authentication protocol provides less authentication delay at the initial stage. As the session time is prolonged the number of user requests for authentication also increases. The load on the authentication server increases. The reauthentication cannot be supported with the expected time of users. So the performance of the EAP based authentication protocol is not suitable for E-Learning. In the proposed CEAP authentication protocol, cache is used to store the user information at initial authentication. When a user requests reauthentication, the user credentials are verified with the cache. Instead of using the authentication for updating the Session Key, ASN gateway performs the key updates with few message transfers. The graph represented in Figure 3 represents the proposed protocol and even when the loads on the system increase the users are served with the minimum delay. The latency for reauthentication in the proposed protocol is reduced by 50% when compared with the EAP based authentication protocol.

### 3.2. Cache Access Latency

**Figure 4:**
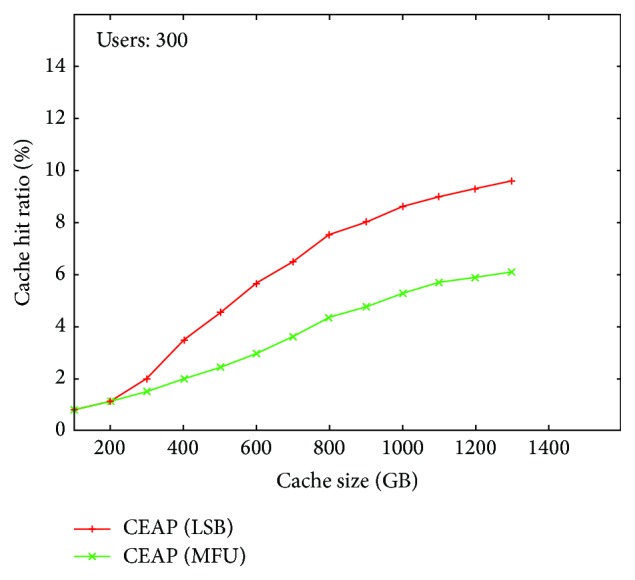
Cache Access Latency.

The latency in authentication is also affected with the time needed to locate the E-Learning user entry in the cache. The user request for reauthentication can be served with the minimum delay only when the user credentials are available in the cache. When a frequent cache miss occurs then the user is requested to perform the full authentication with the authentication server which takes more time for authentication. The cache replacement algorithm plays a major role in providing authentication with minimum latency. The proposed system is implemented with Most Frequently Used Cache Replacement Algorithm and the Least Session Based Cache Replacement Algorithm with different cache sizes (Figure 4).

In the Most Frequently Used Cache Replacement Algorithm, the count is maintained for maintaining the number of times of reauthentication in a particular session. When count exceeds threshold the user entry is selected for replacement. The system implemented with Least Session Based Cache Replacement Algorithm requires that the session with the least time of expiration is evicted and updated with new user entry. In comparison with MFU replacement algorithm, the Least Session Based Cache Replacement Algorithm has better cache hit rate during the reauthentication of E-Learning users.

## 4. Conclusion

In this paper, cache based authentication protocol is used to perform secure reauthentication in WiMAX E-Learning applications. When compared with the existing EAP based authentication protocol, the proposed protocol achieves better performance by reducing the delay occurring during the repeated reauthentication process. The delay is further reduced with the efficient use of Least Session Based Cache Replacement Algorithm.

## Figures and Tables

**Algorithm 1:**
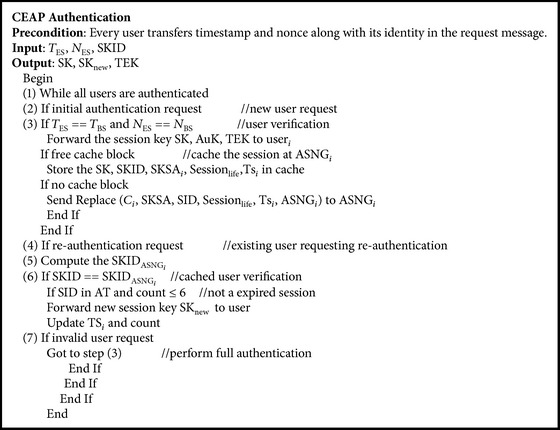
****Proposed Cache Based EAP Authentication Algorithm.

**Algorithm 2:**
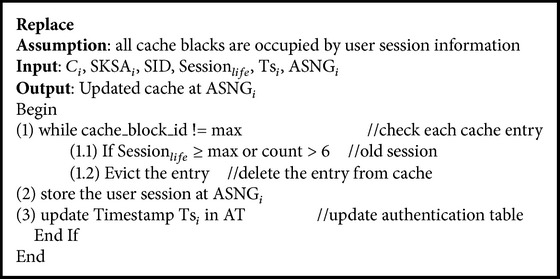
Least Session Based Cache Replacement Algorithm.

**Table 1:** Reauthentication success rate.

*E*[*T*_*n*_]	Fixed session duration, *T* = 3 hrs
10^−3^	0.00419
10^−2^	0.01485
10^−1^	0.136
10^0^	0.6832
10^1^	0.966
10^2^	0.996
